# Genome-wide identification of carbapenem-resistant Gram-negative bacterial (CR-GNB) isolates retrieved from hospitalized patients in Bihar, India

**DOI:** 10.1038/s41598-022-12471-3

**Published:** 2022-05-19

**Authors:** Namrata Kumari, Mukesh Kumar, Amit Katiyar, Abhay Kumar, Pallavi Priya, Bablu Kumar, Nihar Ranjan Biswas, Punit Kaur

**Affiliations:** 1 Department of Microbiology, Indira Gandhi Institute of Medical Sciences, Patna, 800014 Bihar India; 2 Department of Biophysics, All India Institute of Medical Sciences, Ansari Nagar, New Delhi, 110029 India; 3 Bioinformatics Facility, Centralized Core Research Facility, All India Institute of Medical Sciences, Ansari Nagar, New Delhi, 110029 India; 4 Department of Microbiology, Mahavir Cancer Sansthan, Patna, 801505 Bihar India; 5 Department of Pharmacology, Indira Gandhi Institute of Medical Sciences, Patna, 800014 Bihar India

**Keywords:** Antimicrobials, Bacteria, Pathogens, Genome informatics, High-throughput screening

## Abstract

Carbapenemase-producing clinical isolates are becoming more common over the world, posing a severe public health danger, particularly in developing nations like India. Carbapenem-resistant Gram-negative bacterial (CR-GNB) infection has become a fast-expanding global threat with limited antibiotic choice and significant mortality. This study aimed to highlight the carbapenem-resistance among clinical isolates of hospital admitted patients in Bihar, India. A cross-sectional study was conducted with 101 clinical isolates of *Escherichia coli*, *Klebsiella pneumoniae*, *Acinetobacter baumannii*, and *Pseudomonas aeruginosa.* All GNB isolates were tested for their antimicrobial susceptibility using Kirby-Bauer disc diffusion method. Double disc synergy test / modified Hodge test (DDST/MHT) were used to detect carbapenemase production by these isolates. Subsequently, these isolates were evaluated for carbapenem-resistance genes using whole-genome sequencing method. The overall percentage of carbapenem-resistance among GNB was (17/101) 16.8%. The genomic analysis of antimicrobial-resistance (AMR) demonstrates a significantly high prevalence of bla_CTX-M_ followed by bla_SHV_, bla_TEM_, bla_OXA,_ and bla_NDM_ β-lactam or carbapenem resistance genes among clinical isolates of GNB. Co-occurrence of bla_NDM_ with other beta-lactamase-encoding genes was found in 70.6% of carbapenemase-producing isolates. Our study highlights the mechanism of carbapenem-resistance to curb the overwhelming threat posed by the emergence of drug-resistance in India.

## Introduction

Antimicrobial-resistance is a public health issue worldwide, which mainly occurs due to the rough use of antimicrobials^1^. The growing microbial resistance rate to most of the available antimicrobials, including penicillin, cephalosporins, and carbapenem, has made a serious risk to human health^2^. Antimicrobial resistance increases throughout the world and has become very hard to control due to high growth rates of multidrug-resistance among bacteria and lack of consistent observation methods^3^.

The WHO recently emphasized on carbapenemase-producing Gram-negative bacteria (GNB) to be considered critical pathogens^4, 5^. Gram-negative bacteria (GNB), especially *Escherichia coli*, and *Klebsiella pneumoniae* have proven resistant to a wide-ranging variety of antimicrobials accountable for noteworthy mortality all over the world^6, 7^. The development of carbapenem-resistance in GNB is a foremost medical problem, predominantly for immunocompromised patients with serious infections^8^.

Carbapenems are one of the most effective drugs of choice against pathogenic bacteria offering a wide-range of antibacterial activity^9^. This is one of the last options antimicrobials against drug-resistant GNB^10^. The pathogens which are resistant to carbapenem habitually display high intensities of resistance to commonly used antimicrobials. This is not only major cause of high death rates, but also causes prolonged stay of patients in the hospital and high medical expenditures, employing a sensitive, monetary liability on families, particularly in developing poor countries.

Bacteria acquire resistance by multiple mechanisms including enzymatic inactivation, target site mutation, and efflux pumps. Hence, precise identification of AMR in GNB is indispensable for the appropriate administration of apposite antimicrobials. To find AMR in GNB, in vitro cultures are being used to monitor the growth of bacteria for various concentrations of drugs and may need at least 72 hours to acquire precise antimicrobial susceptibility results. On the other hand, advancement in whole-genome sequencing (WGS) has reinforced the evaluation of the complete DNA sequence of bacteria. WGS delivers a vital description of the genotype of an individual organism. It may contribute to many ways to tackle AMR. WGS data can give mechanistic insight into the antimicrobial-resistance for even those drugs not being tested routinely^11^.

India is a prime location for AMR pathogens because of the overuse of antimicrobials and so is Bihar. However, to the best of our knowledge no data is available on carbapenem-resistance genes from the Bihar region. Hence, this study aimed to genotypically characterize the carbapenemase-producing clinical GNB isolates obtained from in-patients at I.G.I.M.S., Patna, India.

## Methods

### Study design

A cross-sectional investigation was conducted on CR-GNB strains isolated from routine clinical samples of hospital admitted patients coming to the microbiology laboratory of Indira Gandhi Institute of Medical Sciences, Patna, Bihar for a duration of 10 months from March 2019 to December 2019. All of the methods followed the guidelines set out by the Clinical and Laboratory Standards Institute (CLSI). The lab work and data analysis were completed at Indira Gandhi Institute of Medical Sciences (I.G.I.M.S), Patna, and All India Institute of Medical Science (A.I.I.M.S.), New Delhi, respectively. The study was reviewed and approved by the ethical committee of IGIMS, Patna, India (451/IEC/2018/IGIMS). Written informed consent was taken from the participants.

### Bacterial isolates

Gram-negative bacteria (GNB) including *E. coli*, *K. pneumoniae*, *A. baumannii*, and *P. aeruginosa* were isolated and identified by standard manual conventional method from the culture of the routine clinical samples like blood, vascular catheter tip, urine, bile, ascitic fluid, pus, sputum, endotracheal tube-aspirate, and broncho-alveolar lavage (BAL) fluid. The samples were inoculated on blood agar (Himedia M073), MacConkey agar (Himedia M008), and Nutrient agar (Himedia M001). Blood and respiratory samples were inoculated also on chocolate agar (Himedia M103). After overnight incubation at 37 °C, Gram stain (Himedia K001) was performed from the growth on the plates. Biochemical tests were performed on the isolates which were gram-negative bacilli. The biochemical tests like catalase test (3% H2O2), oxidase test (Himedia GRM445), indole test (Kovac’s reagent, Himedia R008), citrate utilization test (Simmon’s citrate media, Himedia M099), urea hydrolysis test (Christensen's urea agar base Himedia M112), motility test, triple sugar iron test (Himedia M021), decarboxylase test (Himedia Moeller decarboxylase broth W/lysine HCL/ornithine HCl/arginine HCl M687/M688/M689), methyl red, Voges-Proskauer test (Himedia M070S), Hugh and Leifson OF test (Himedia M826) and nitrate reduction test (Nitrate broth, Himedia M439S) were performed as per need for identification of *Escherichia coli*, *Klebsiella pneumoniae*, *Pseudomonas aeruginosa,* and *Acinetobacter baumannii*. All the media and reagents used were from HiMedia Laboratories Pvt. Ltd, Mumbai, India.

### Antimicrobial susceptibility testing of GNB isolates

Antimicrobial susceptibility testing of isolates was performed using the standard Kirby-Bauer Disc Diffusion Method. For quality control, suitable ATCC control strains were used (CLSI, M100 doc., ed. 28th). A broth culture of the isolate with turbidity equivalent to McFarland 0.5 turbidity standard was lawn cultured over the Mueller Hinton agar plate (Himedia M173) and allowed to dry. Then the antimicrobial discs were applied and incubated overnight at 35 °C ± 2 °C. Sensitivity to the drug was determined by the zone of inhibition of bacterial growth around the disc. The following antimicrobials (Hi-Media disc in mcg) were tested: ampicillin (10 mcg), amoxicillin clavulanic acid (20/10 mcg), cefotaxime (30 mcg), ceftriaxone (30 mcg), ceftazidime (30 mcg), piperacillin-tazobactum (100/10 mcg), sulfamethoxazole-trimethoprim (25 mcg) nitrofurantoin (100 mcg), aztreonam (30 mcg), ciprofloxacin (5 mcg), gentamicin (10 mcg), amikacin (30 mcg), minocycline (30 mcg), meropenem (10 mcg) and imipenem (10 mcg). The Clinical and Laboratory Standards Institute (CLSI) 2018 standards were used to quantify and interpret zone diameter. Antimicrobial discs used were from HiMedia Laboratories Pvt. Ltd, Mumbai, India.

### Detection of carbapenemase production

Carbapenem-resistance screening of GNB isolates was done using meropenem (10ug) disc. Modified Hodge test (MHT) and meropenem double disc synergy test (DDST, employing EDTA disc) were used to validate phenotypic identification of carbapenemase production in these resistant (meropenem disc screened) isolates.

### Modified Hodge test (MHT) for phenotypic detection of carbapenemase production

According to CLSI^12^ guidelines, all isolates were subjected to the modified Hodge test. A lawn culture of the 1:10 dilution of *Escherichia coli* ATCC 25922 was carried out on Mueller Hinton agar plate and a 10-mcg meropenem susceptibility disk was placed in the center of the test area. A straight line was then drawn from the edge of the disk to the edge of the plate with the test organism. A total of four strains were tested on the same plate with one disk and incubated overnight at 35 °C ± 2 °C. An interpretation was done after 16–24 h of incubation. Positive modified Hodge test showed a clover leaf-like indentation of the *Escherichia coli* 25922 strain growing along with the test organism growth streak within the disk diffusion zone indicating production of carbapenemase and a negative test showed no growth of the *Escherichia coli* ATCC 25922 along the test organism growth streak within the disk diffusion zone.

### Meropenem-EDTA double disc synergy test (DDST)

The double-disc synergy test of Meropenem and EDTA^13–15^ was done to screen metallo-β-lactamase-producing strains of *Pseudomonas* and *Acinetobacter* species. A lawn culture of the organism was inoculated onto MHA plate as per CLSI guidelines. To prepare a 0.5 Mcfarland standard of the isolate to be tested, two to three colonies were inoculated onto peptone water and incubated for two to three hours at 37 °C. Then a cotton swab which was sterile attached on a wooden stick was dipped into the 0.5 McFarland standard inoculum. In order to release excess fluid, the soaked swab was firmly rotated against the inner wall of the tube. The swab was used to streak the agar surface of the entire MHA plate three times, rotating the swab at an angle of 60 °C between each streaking. After drying, a 10 μg meropenem disc was placed on the lawn culture with a distance of 20 mm centre to centre from a blank disc. To achieve the desired concentration of 750 μg, 10 μl of McFarland EDTA was added to the blank disc and incubated at 37 °C for 16 to 18 h. If there was an enhancement in the inhibition zone of > 5 mm in the area between meropenem disc and the EDTA disc in comparison with the zone of inhibition on the far side of the drugs, was interpreted as positive results.

Isolate positive by either or both the tests were taken as phenotypically detected carbapenemase producer. We maintained all phenotypically detected isolates at − 80 °C in nutrient broth containing 7.5% (v/v) glycerol. A sub-culture of these carbapenem-resistant isolates was performed on blood agar, followed by whole-genome sequencing. All the media and reagents used were from HiMedia Laboratories Pvt. Ltd, Mumbai, India.

### Whole-genome sequencing

Genomic DNA of pathogens was isolated from freshly sub-cultured colonies of carbapenem-resistant bacteria using DNeasy Blood and Tissue Kit (Qiagen, Catalogue # 69505), following the manufacturer's recommendations. DNA concentration of the purified DNA was calculated on Qubit (Thermo Fischer Scientific), using Qubit™ 1X dsDNA HS Assay Kit (Invitrogen, Catalogue # Q33231). The genomic DNA was stored at − 20 °C. The Illumina MiSeq (Illumina, San Diego, CA, 107 USA) technology was used to sequence these DNA isolates. Using the Nextra DNA Flex Library Prep Kit, a MiSeq short-read sequencing library was created with 1 ng pure DNA (Illumina, Cat. No. 20018704). Bead-linked transposomes (BLTs) were employed in this kit to facilitate simultaneous DNA fragmentation and Illumina sequencing primer tagging (Tagmentation). These sequencing-ready DNA fragments were amplified by PCR and indexes (for sample identification) and adapters were further added to them (using Nextera DNA CD Indexes, Cat. No.20018707). For each library, the normalization was performed to bring them to a standard concentration of 4 nM. Sequencing ready fragments were further washed and pooled. The average size of the libraries was found to be around 550 bp which was calculated with the help of Agilent Bioanalyzer 2100 using High Sensitivity DNA Assay Kit (5067-4626). The libraries were next loaded onto the sequencer Illumina MiSeq 500 platform for sequencing by synthesis/bridge amplification. Casava (v.1.8.2) was used to de-multiplex the output data files and convert them to FASTQ files (Illumina, Inc, USA).

### Preprocessing and de-novo assembly

FastQC-0.11.9 (https://www.bioinformatics.babraham.ac.uk) was used to assess the read quality. Trimmomatic-0.39 was used to trim adapters and low-quality sequences^16^. Velvet was used to build contigs using clean reads^17^. QUAST^18^ was used to evaluate the assembled genome's quality. Prokka^19^ (v1.12) was used to annotate the assembled bacterial genomes. Under the accession number PRJNA744890, the sequencing SRA data were submitted to the National Center for Biotechnology Information (http://www.ncbi.nlm.nih.gov/).

### Detection of resistance genes

The RGI-CARD (Comprehensive Antibiotic Resistance Database) and Pathogenwatch (Center for Genomic Pathogen Surveillance) were used to predict the resistance genes in the assembled Gram-negative bacteria genomes. We utilized 50 percent sequence identity and 70 percent query coverage as cut-off criteria. The acquired antimicrobial resistance genes and genes associated with chromosomal point mutations were identified using the ResFinder webserver 3.0 (https://cge.cbs.dtu.dk/services/ResFinder/).

### Ethics statement

Written informed consent was taken by the participants and the study was reviewed and approved by the ethical committee of IGIMS, Patna, India (451/IEC/2018/IGIMS).

## Results

Out of 101 Gram-negative bacteria (GNB) isolated from different clinical samples, 17 (16.8%) were carbapenemase producers (Table 1a and b).

**Table 1:** Distribution and phenotypic carbapenem-resistance rate of isolated Gram-negative bacteria using MHT and DDST methods.

(a) Organism-wise distribution of CR isolates			
	No of isolates	No of CR isolates	Percentage (%)
*E. coli*	71	9	12.7
*K. pneumoniae*	12	5	41.7
*P. aeruginosa*	11	2	18.2
*A. baumannii*	7	1	14.3
	Total = 101	Total = 17	Total % = 16.8

(b) Specimen-wise distribution of CR isolates			
	No of isolates	Carbapenem-resistance rate (%)	Positive by
Blood and vascular catheter tip	6	*K. pneumoniae*—1/06 (16.7%)	MHT and DDST
		*A. baumannii*—1/06 (16.7%)	MHT and DDST
		*E. coli*—1/06 (16.7%)	MHT and DDST
Pus and body fluids	36	*E. coli* –2/36 (5.6%)	MHT and DDST
		*K. pneumoniae*—1/36 (2.8%)	MHT and DDST
Urine	49	*K. pneumoniae*—2/49 (4.1%)	1-MHT and DDST 1-MHT only
		*E. coli*—5/49 (10.2%)	3-MHT and DDST 2-MHT only
		*P. aeruginosa*—1/49 (2.0%)	MHT and DDST
Lower respiratory samples (Endotracheal aspirate, Broncho-alveolar Lavage, Sputum)	10	*K. pneumoniae*—1/10 (10.0%)	MHT and DDST
		*E. coli*—1/10 (10.0%)	MHT and DDST
		*P. aeruginosa*—1/10 (10.0%)	DDST only
	Total = 101	Total = 17, positive by MHT / DDST	

*DDST double disc synergy test, *MHT* modified hodge test.

### Demographic distribution

The isolates found were from different age group patients ranging from 6 to 76 years old. Isolates from males 76.5% (13/17) were more in number as compared to isolates from female 23.5% (4/17) inpatients. Maximum numbers of these carbapenemase-producing isolates were found from medicine ICU 47.0% (8/17) followed by surgery ward 35.3% (6/17) admitted patients. Carbapenemase-producing isolates were found in the following clinical specimens: blood 11.8% (2/17), vascular catheter tip 5.9% (1/17), urine 47.1% (8/17), bile 5.9% (1/17), pus 11.8% (2/17), sputum 5.9% (1/17), endotracheal tube aspirate 5.9% (1/17) and BAL 5.9% (1/17) fluid. Urine samples had the highest number of carbapenem-resistant isolates. Sample-wise distribution and phenotypic carbapenem-resistance rate of isolated GNB are given in Table 1b.

### Antimicrobial susceptibility and phenotypic screening

Table 2 shows the phenotypic antimicrobial resistance pattern of isolated GNB. For 101 GNB isolates tested, the highest percentage of resistance was recorded in ampicillin (95.8%) followed by ciprofloxacin (94.4%), amoxicillin-clavulanic acid (89.3%), cefotaxime (88.8%), ceftriaxone (84.0%), piperacillin-tazobactum (78.9%), ceftazidime (76.2%), tobramycin (66.7%), sulfamethoxazole-trimethoprim (66.2%), gentamicin (60.7%) and amikacin (30.3%). Meropenem and imipenem both had a 22.1% resistance rate. The percentage of bacterial resistance to carbapenems was highest in *K. species*. Amikacin showed good sensitivity in GNB among aminoglycosides.

**Table 2:** Antimicrobial-resistance (Kirby-Bauer Disc Diffusion) rates (%) of isolated Gram-negative bacteria.

Antimicrobials (n = 101)	*E. coli* (n = 71)	*K. pneumoniae (*n = 12)	*A. baumannii (n* = *7)*	*P. aeruginosa (n* = *11)*
Ampicillin	68 (95.8%)	11 (91.7%)	7 (100%)	–
Amoxycillin-clavulanic acid	60 (84.5%)	10 (83.3%)	7 (100%)	–
Cefotaxime	59 (83.1%)	10 (83.3%)	7 (100%)	–
Ceftriaxone	59 (83.1%)	10 (83.3%)	6 (85.7%)	–
Ceftazidime	57 (80.3%)	9 (75.0%)	6 (85.7%)	7 (63.6%)
Piperacillin-tazobactum	59 (83.1%)	10 (83.3%)	6 (85.7%)	7 (63.6%)
Sulfamethoxazole-trimethoprim	43 (60.6%)	9 (66.7%)	5 (71.4%)	–
Aztreonam	–	–	–	9 (81.8%)
Ciprofloxacin	65 (91.5%)	11 (91.7%)	7 (100%)	–
Ofloxacin	–	–	–	8 (72.7%)
Gentamicin	35 (49.3%)	7 (58.3%)	5 (71.4%)	7 (63.6%)
Amikacin	19 (26.8%)	4 (33.3%)	3 (42.8%)	2 (18.2%)
Tobramycin	49 (69.0%)	8 (66.7%)	6 (85.7%)	5 (45.4%)
Minocycline	–	–	1 (14.3%)	–
Meropenem	10 (14.1%)	5 (41.7%)	1 (14.3%)	2 (18.2%)
Imipenem	10 (14.1%)	5 (41.7%)	1 (14.3%)	2 (18.2%)

### Prevalence and distribution of beta-lactamase genes

**Figure 1:**
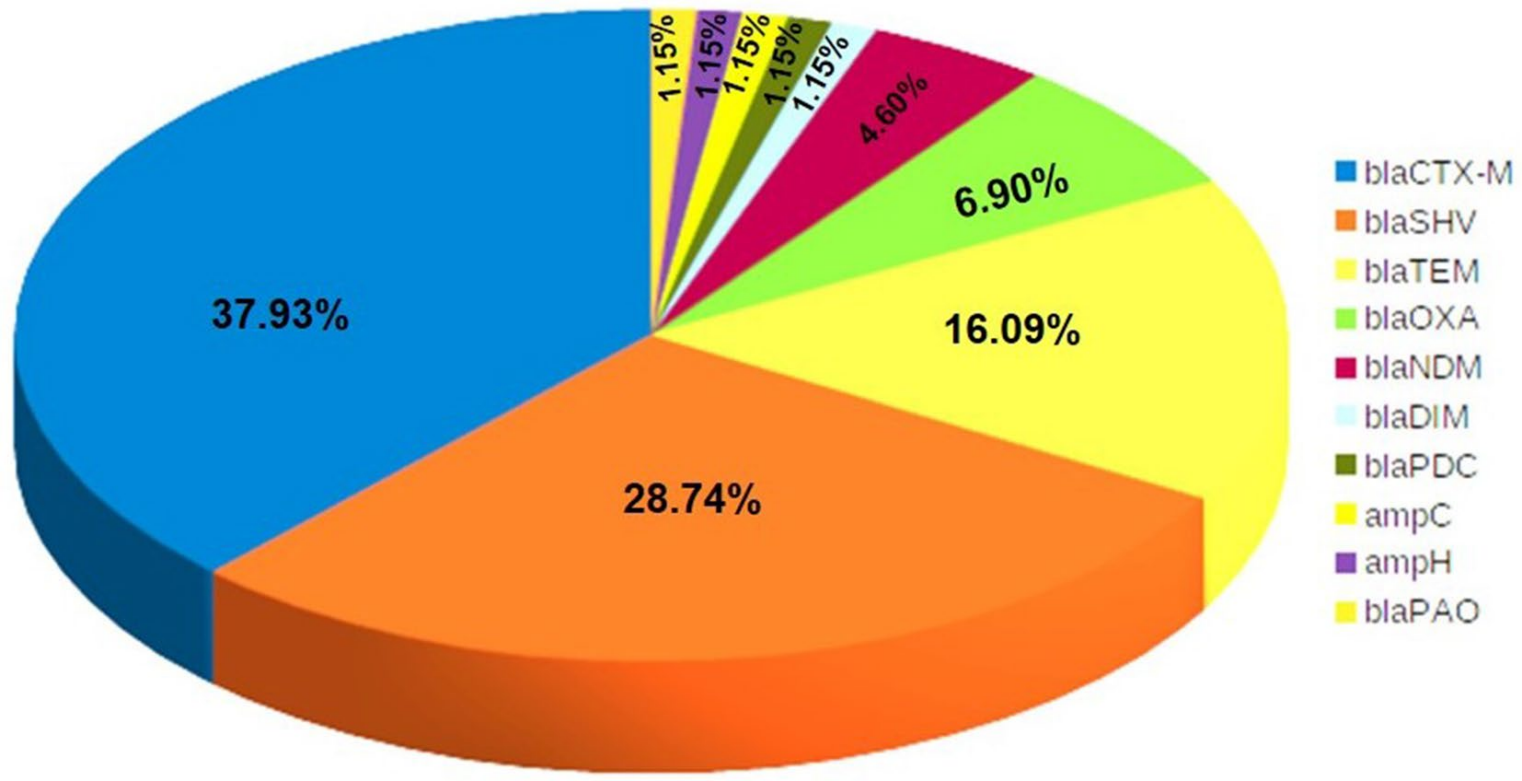
Types of carbapenem-resistance gene in Gram-negative bacteria.

**Figure 2:**
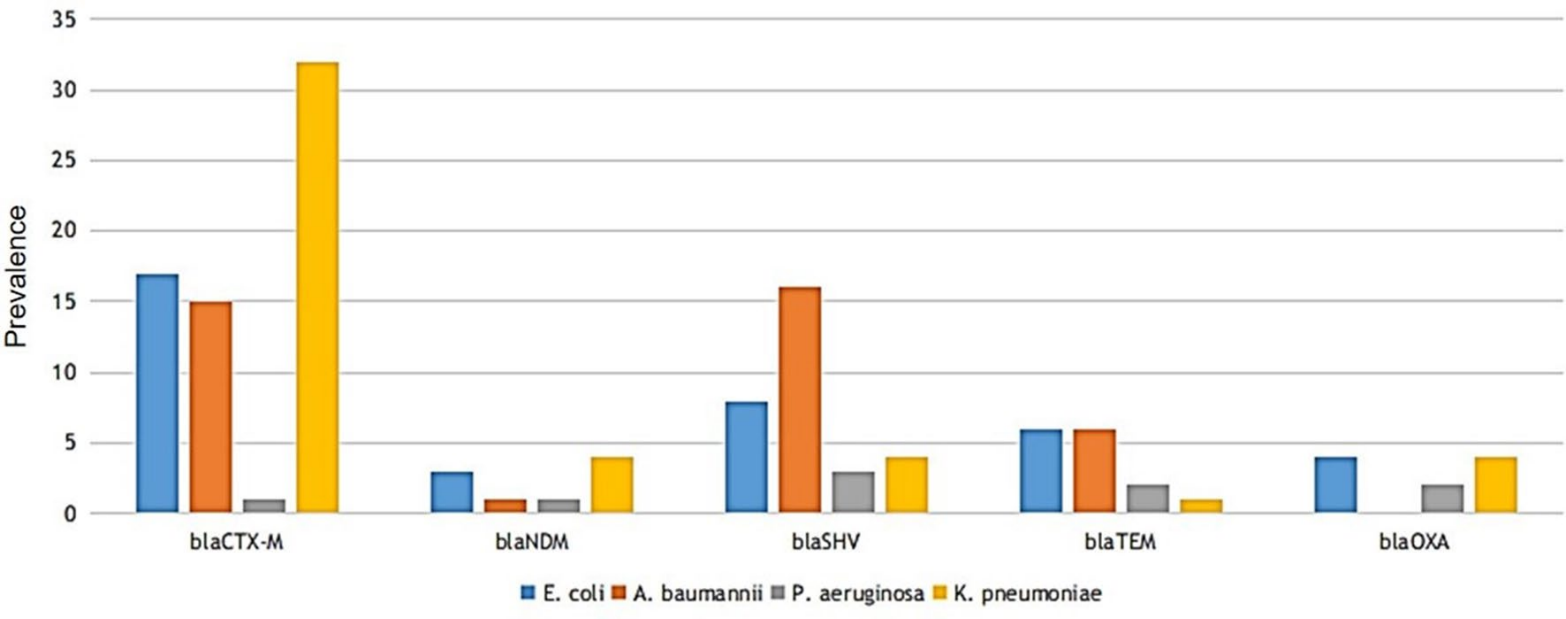
Isolates-wise prevalence and distribution of carbapenemase genes in Gram-negative bacteria.

The 17 Gram-negative bacterial isolates screened positive for carbapenemase by MHT and DDS phenotypic method were further confirmed genotypically using antimicrobial repositories (such as CARD, Pathogenwatch, and ResFinder), yielding 87 types of beta-lactamase**/**carbapenemase genes. Among them, 37.9% types of genes were bla_CTX-M_ followed by bla_SHV_ (28.7%), bla_TEM_ (16.1%), and bla_OXA_ (6.9%) (Fig. 1; Supplemental Table S1). Analysis revealed that bla_CTX-M-15_, bla_NDM-5_, bla_TEM-1D_, and bla_OXA-10_ were the most frequent subtype in their respective groups of GNB isolates (Supplemental Fig. S1). Carbapenemase including subtypes bla_NDM_ (bla_NDM-1_, bla_NDM-5_, bla_NDM-16,_ and bla_NDM-20_), bla_OXA_ (bla_OXA-23_, and bla_OXA-181_) and bla_DIM_ (bla_DIM-1_) were observed which makes bacteria resistant to a broad range of carbapenem antimicrobials. The strain-wise prevalence of carbapenemase was bla_NDM_ (12/17) including subtypes bla_NDM-1_(3/17)_,_ bla_NDM-5_ (8/17)_,_ bla_NDM-16_ (1/17)_,_ bla_NDM-20_ (2/17), followed by bla_OXA_ (4/17) including subtypes bla_OXA-23_ (1/17)_,_ bla_OXA-181_ (3/17), and bla_DIM-1_ (2/17). Similarly, the prevalence of beta-lactamase was bla_CTX-M_ (13/17), followed by bla_OXA_ (8/17) including subtypes bla_OXA-1_ (1/17), bla_OXA-10_ (6/17), bla_OXA-50_ (1/17), bla_OXA-488_ (1/17), bla_SHV_ (5/17) including subtypes bla_SHV-11_ (4/17), bla_SHV-148_ (1/17), and bla_TEM_ (7/17) including bla_TEM-104_ (1/17), bla_TEM-1d_ (6/17). In addition, colistin-resistance genes (MgrB/PmrB) were also observed in *E. coli, K. pneumoniae* and *P. aeruginosa* strains. Table 3 and Supplementary Table S2 provide a genotypic description of the antimicrobial-resistance genes of all 17 isolates. The gene occurrence of bla_NDM_ and bla_OXA_ was dominantly observed in *K. pneumoniae*, followed by *E. coli* whereas bla_CTX-M_ was mainly found in *K. pneumoniae*, followed by *E. coli* (Fig. 2 and Supplemental Table S3).

**Table 3:** Genotype profiling of 17 beta-lactam/carbapenem-resistant Gram-negative bacteria (CR-GNB).

*Strain	Beta-lactams/ESBLs											Carbapenem							Colistin	
	AmpC1	PDC-10	SHV-11	SHV-148	TEM-104	TEM-1D	OXA-1	OXA-10	OXA-50	OXA-488	CTX-M15	DIM-1	NDM-1	NDM-5	NDM-16	NDM-20	OXA-23	OXA-181	MgrB	PmrB
AB01	0	0	0	0	0	0	0	0	0	0	0	0	0	1	0	0	0	0	0	0
EC02	1	0	0	0	0	0	0	1	0	0	1	0	1	0	1	0	0	1	0	0
EC03	0	0	0	0	0	1	0	0	0	0	1	0	0	1	0	0	0	0	0	0
EC04	0	0	1	0	0	0	0	0	0	0	0	0	0	0	0	0	0	0	0	0
EC05	0	1	1	0	0	1	0	1	0	1	1	1	0	1	0	0	0	0	0	0
EC06	0	0	0	1	0	1	0	0	0	0	1	0	0	1	0	0	0	1	0	0
EC07	0	0	1	0	0	1	0	1	0	0	1	0	0	1	0	0	0	0	0	1
EC08	1	0	0	0	0	0	0	1	0	0	0	0	1	0	0	0	0	0	0	0
EC09	0	0	0	0	0	0	0	0	0	0	0	0	0	0	0	0	0	0	0	0
EC10	0	0	1	0	0	1	0	1	0	0	0	0	0	1	0	0	0	0	0	0
KP11	0	0	0	0	0	1	0	0	0	0	0	0	0	0	0	0	0	0	0	1
KP12	0	0	0	0	0	0	0	0	0	0	0	0	0	0	0	1	0	0	1	1
KP13	0	0	0	0	0	0	0	0	1	0	0	1	0	0	0	0	0	0	0	0
KP14	1	0	0	0	0	0	0	0	0	0	0	0	0	1	0	0	0	0	0	0
KP15	1	0	0	0	0	0	0	1	0	0	1	0	1	0	0	1	0	1	0	0
PA16	1	0	0	0	1	0	1	0	0	0	0	0	0	1	0	0	1	0	0	0
PA17	0	0	0	0	0	0	0	0	0	0	1	0	0	0	0	0	0	0	0	1

*AB Acinetobacter baumannii, *EC* Escherichia coli, *PA* Pseudomonas aeruginosa, *KP* Klebsiella pneumonia, 1: present, 0: absent.

### Co-existence genes

It has been observed that the majority of GNB isolates had more than one beta-lactamase/carbapenemase gene, where co-existence genes were mostly found in *E. coli* and *K. pneumoniae* isolates. Among, co-existence of two genes namely “bla_CTX-M_ + bla_NDM_” (1/17), and “bla_CTX-M_ + bla_SHV_” (1/17), was observed in *K. pneumoniae* and *E. coli,* respectively, whereas co-existence of three genes “bla_CTX-M_ + bla_NDM_ + bla_OXA_” was commonly observed in both *K. pneumoniae* and *E. coli.* Likewise, “bla_CTX-M_ + bla_TEM_ + bla_OXA_” and “bla_CTX-M_ + bla_NDM_ + bla_SHV_” pattern of three genes was found in *K. pneumonia*, whereas “bla_CTX-M_ + bla_NDM_ + bla_TEM”_ co-existence genes pattern was found exclusively in *E. coli.* Co-existence of four genes namely *“*bla_CTX-M_ + bla_NDM_ + bla_SHV_ + bla_TEM_” was detected in 3 species (*K. pneumoniae, E. coli* and *A. baumannii*), whereas “bla_CTX-M_ + bla_NDM_ + bla_OXA_ + bla_TEM_” was observed in *E. coli* only. Interestingly, co-existence of five genes “bla_CTX-M_ + bla_NDM_ + bla_SHV_ + bla_OXA_ + bla_TEM_” was found in 3 isolates of *E. coli.* A high co-existence rate in GNB may provide further insight into the epidemiology of resistance acquisition. Table 4 shows the distribution of co-existence genes among different Gram-negative bacteria. Analysis revealed beta-lactamases co-existence genes with bla_NDM_ in 12 (70.6%) of carbapenemase-producing isolates.

**Table 4:** Co-existence genes conferring *resistance* to *β-lactams* carbapenem drugs in Gram-negative bacteria.

β-lactamases genes	*E. coli* (n = 8/9)	*K. pneumonia* (n = 5/5)	*P. aeruginosa* (n = 0/2)	*A. baumannii* (n = 1/1)
CTX-M + NDM	0	1	0	0
CTX-M + SHV	1	0	0	0
CTX-M + NDM + OXA	1	1	0	0
CTX-M + TEM + OXA	0	1	0	0
CTX-M + NDM + SHV	0	1	0	0
CTX-M + NDM + TEM	1	0	0	0
CTX-M + NDM + OXA + TEM	1	0	0	0
CTX-M + NDM + SHV + TEM	1	1	0	1
CTX-M + NDM + SHV + OXA + TEM	3	0	0	0

## Discussion

Gram-negative bacterial (GNB) infections that produce carbapenemase are on the rise worldwide, including in India^20^. As of today, carbapenems are the preferred medicine for treating serious hospital-acquired infections. Recent studies have shown a very high carbapenem resistance in India and the Indian subcontinent, which necessitates the use of alternative treatments. It would be interesting to precisely identify carbapenemase-producing microbes, and this would require phenotypic and genotypic studies to identify all carbapenemase-producing genes. This study was carried out in 1060 bedded super-specialty tertiary care hospital in Bihar, India. The majority of patients were referred after using antimicrobials. In addition, 47.0% of the isolates in the study were from the intensive care unit, where patients are more prone to undergo invasive procedures. These factors and prolonged hospital stay may have contributed to the high prevalence of carbapenem-resistant in admitted patients. Carbapenemase activity has been known in β-lactamases classes namely A, B, and D^21–24^. The prevalence of CR-GNB in the current study was 16.8% as compared to the 31.8% in Western Maharashtra^23^. Similarly, Wattal et al. found a prevalence rate of 13 to 51.0% in a tertiary care hospital in Delhi^24^. Nair et al. found it to be around 12.3% in a study in Mumbai^8^, while Gupta et al. found it to be between 17.0 and 22.0% in a study in northern India^25^.

The prevalence of CTX-M-15 observed in our study is consistent with the findings from other parts of India. For example, study conducted on 130 clinical samples in *E. coli* and *K. pneumoniae* taken from Aligarh, Varanasi (Uttar Pradesh; North India) and Hubli (Karnataka; South India) have shown the prevalence of bla_CTX-M-15_ gene^8^. In another study, 300 isolates of *E. coli* were tested and found that bla_CTX-M-15_ was the most dominant gene^26^. Likewise, the study conducted on carbapenem-resistance genes in urinary isolates of *K. pneumoniae* (from Southern India) showed a high prevalence of bla_CTX-M-15_ gene^27^, where's an analysis of 1275 strains from *E. coli* and *K. pneumoniae* showed the increasing prevalence of bla_CTX-M-15_ gene in the patients from the rural community of North India^28^.

Similar trends for bla_CTX-M-15_ genes were observed in neighboring countries including Nepal^3^, Bangladesh^29^, Brazil^30^, China^31^, Pakistan^32^, Ethiopia^33^, Switzerland^34, 35^, Argentina^36^, Netherlands^37^, Japan^38^ and United States in GNB isolates. For example, the study conducted in Ethiopia showed the prevalence of bla_CTX-M-15_ type extended-spectrum β-lactamases in *E. coli* (92.3%) and *K. pneumoniae* (96.7%) among clinical isolates of GNB^33^. In another study, ESBL-producing *E. coli* contained a higher prevalence of bla_CTX-M-15_ (58.4%) gene in patients admitted at the hospital, Kathmandu, Nepal^39^. The abundance of bla_CTX-M-15_ gene was also observed in *E. coli* clinical isolates from the community and hospital-based infection in China^31^. High prevalence of ESBL-encoding bla_CTXM-15_ gene was observed in 2372 clinical samples of GNB including *E. coli, K. pneumoniae, P. aeruginosa, Enterobacter spp*. and *A. baumannii* obtained from the hospitals and diagnostic research center of Lahore, Pakistan^8^. The carbapenemase activity for bla_CTX-M-15_ has been reported earlier^40, 41^.

The abundance of bla_CTX-M_ genes in different species suggests horizontal gene transfer is occurring now or in the past. For example, *E. coli* from healthy food animals can be key repositories of beta-lactamase genes and may contribute to the spread and transmission of these beta-lactamase genes, and lateral transfer of resistance genes between animals and humans. In contrast, bla_NDM_ and bla_OXA-181_ were observed to be highly prevalent in GNB isolates in Tamil nadu^42^ as well as Mumbai^43^. Likewise, bla_VIM_^44^, and bla_IMP_^45^ was observed to be the most common gene in CR-GNB isolates. We found 3 types of carbapenemase namely bla_NDM_ (4.6%), bla_OXA_ (2.3%), and bla_DIM_ (1.6%) in our study. Though these carbapenemase gene are not common, it is concerning because it can be resistant to even more antimicrobials. Among, subclass B1 metallo-beta-lactamase (bla_NDM_), higher prevalence of bla_NDM-5_ was detected in GNB isolates which may confer higher resistance against carbapenems than bla_NDM-1_ as reported earlier^46^. Varying geographic locations, different levels of healthcare institutions engaged, different levels of exposure to healthcare environments, antibiotic use, and antibiotic stewardship procedures may all contribute to these disparities.

Our study found multiple co-existence genes within the same isolate, where beta-lactamase-encoding co-existence genes with bla_NDM_ were found in 70.6% of carbapenemase-producing isolates. This creates a new challenge for the treatment of infections caused by carbapenem-resistant strains because carbapenem-resistant genes could co-exist with beta-lactamases and other resistant genes on plasmids^2, 47^. In addition to this, co-existence to carbapenem retains genes that make them resistant to other antimicrobials, which threatens global antibiotic chemotherapy, patients recovery, and the economy^7, 48, 49^. The carbapenem resistance can be caused by the presence of bla_NDM_, bla_CTX-M_, bla_TEM_, bla_SHV_, and bla_OXA_ as well as impermeability^50^. This is particularly problematic in India, where beta-lactamase/carbapenemase prevalence is relatively high. In our study, more than half of the isolates showed multidrug resistance (MDR) to the most common antimicrobials, suggesting that carbapenemase-encoding genes can serve as an index of phenotype in CR-GNB isolates. The acquisition and horizontal transfer of resistant genes from a variety of sources, including pathogenic bacteria, the environment, and animals, could be the main causes of resistance's uncontrolled expansion^51^. Poor infection management in the country might be another reason for the high incidence of MDR and the acquisition of resistance genotypes, necessitating immediate action to combat the burgeoning AMR.

## Conclusions

Carbapenemase-producing bacteria were detected in abundance in the Bihar region. The present study highlights the overwhelming threat of carbapenem-resistance in GNB. A high co-existence rate in multidrug-resistant GNB was observed which may provide further insight into the epidemiology of resistance acquisition. The prevalence of carbapenemase-encoding genes (bla_NDM_, bla_OXA,_ and bla_DIM)_ found from this study is a rising threat in India which requires immediate attention from the healthcare perspective. Therefore, a strict policy to prevent the misuse of antimicrobials should be imposed to control the drug-resistance in India.

## Supplementary Information

Supplementary Information.

## Data Availability

Whole-genome sequences have been deposited in the NCBI under the accession number PRJNA744890.

## Abbreviations

CR-GNB: Carbapenem-resistant Gram-negative bacterial
CLSI: Clinical and Laboratory Standards Institute
MHT: Modified Hodge test
DDST: Double disc synergy test
CARD: Comprehensive Antibiotic Resistance Database
AMR: Antimicrobial resistance
CTX-M: Cefotaxime-hydrolyzing β-lactamase–Munich
NDM: New Delhi metallo-β-lactamase
OXA: Oxacillin carbapenemase/oxacillinase
SHV: Sulfhydryl variant of the TEM enzyme
TEM: Temoneira class A extended-spectrum β-lactamase
VIM: Verona integron-encoded metallo-β-lactamase, metallo-βlactamases (MBLs)
