# Supplementary material for: Genome-wide identification of carbapenem-resistant Gram-negative bacterial (CR-GNB) isolates retrieved from hospitalized patients in Bihar, India
Source: Sci Rep. 2022 May 19;12:8477. doi: 10.1038/s41598-022-12471-3 (PMC9120164; doi:10.1038/s41598-022-12471-3)

Table S1: Type of β-lactams carbapenem-resistance genes in Gram-negative bacterial isolates by CARD, Pathogenwatch and Resfinder

**bla_{CTX-M}**	**bla_{SHV}**	**bla_{TEM}**	**bla_{OXA}**	**bla_{NDM}**	**bla_{DIM}**	**bla_{PAO}**	**bla_{PDC}**	**ampC**	**ampH**
blaCTX-M-103	blaSHV-106	blaTEM-1	blaOXA-1	blaNDM-16	blaDIM-1	blaPAO	blaPDC-12	ampC1	ampH
blaCTX-M-114	blaSHV-11	blaTEM-104	blaOXA-10	blaNDM-20					
blaCTX-M-139	blaSHV-12	blaTEM-141	blaOXA-181	blaNDM-5					
blaCTX-M-142	blaSHV-129	blaTEM-198	blaOXA-23	blaNDM-1					
blaCTX-M-15	blaSHV-13	blaTEM-1A	blaOXA-488						
blaCTX-M-150	blaSHV-142	blaTEM-1B	blaOXA-50						
blaCTX-M-156	blaSHV-148	blaTEM-206							
blaCTX-M-163	blaSHV-15	blaTEM-209							
blaCTX-M-164	blaSHV-154	blaTEM-214							
blaCTX-M-167	blaSHV-155	blaTEM-216							
blaCTX-M-170	blaSHV-160	blaTEM-217							
blaCTX-M-176	blaSHV-161	blaTEM-234							
blaCTX-M-180	blaSHV-172	blaTEM-30							
blaCTX-M-182	blaSHV-185	blaTEM-84							
blaCTX-M-183	blaSHV-28								
blaCTX-M-184	blaSHV-31								
blaCTX-M-186	blaSHV-55								
blaCTX-M-188	blaSHV-61								
blaCTX-M-193	blaSHV-66								
blaCTX-M-194	blaSHV-67								
blaCTX-M-202	blaSHV-78								
blaCTX-M-203	blaSHV-86								
blaCTX-M-208	blaSHV-94								
blaCTX-M-209	blaSHV-96								
blaCTX-M-210	blaSHV-98								
blaCTX-M-216									
blaCTX-M-22									
blaCTX-M-3									
blaCTX-M-33									
blaCTX-M-55									
blaCTX-M-71									
blaCTX-M-82									
blaCTX-M-88									

Table S2: Antimicrobial-resistance profile of 17 carbapenem-resistance Gram-negative bacteria (CR-GNB)

*Strain	CTX-M variants	TEM variants	OXA variants	NDM variants	SHV variants	DIM variants	PDC variants	PAO variants
AB01	1	1	0	1	1	0	0	0
EC02	1	0	1	1	0	0	0	0
EC03	1	1	0	1	1	0	0	0
EC04	1	0	0	0	1	0	0	0
EC05	1	1	1	1	1	1	1	1
EC06	1	1	1	1	1	0	0	0
EC07	1	1	1	1	1	0	0	0
EC08	1	1	1	1	0	0	0	0
EC09	0	0	0	0	0	0	0	0
EC10	0	1	1	1	1	0	0	0
KP11	1	0	1	0	0	0	0	0
KP12	1	0	0	1	1	0	0	0
KP13	1	0	1	0	0	1	0	1
KP14	1	0	0	1	0	0	0	0
KP15	1	0	1	1	0	0	0	0
PA16	0	1	1	0	1	0	0	0
PA17	1	0	0	0	0	0	0	0

***AB:** Acinetobacter baumannii; **EC:** Escherichia coli; **PA:** Pseudomonas aeruginosa; **KP:** Klebsiella pneumonia; **1:** present; **0:** absent

Table S3: Distribution of β-lactams carbapenem-resistance genes in E. coli, A. baumannii, P. aeruginosa, and K. pneumoniae

	blaCTX-M	blaNDM	blaSHV	blaTEM	blaOXA
E. coli	17	3	8	6	4
A. baumannii	15	1	16	6	0
P. aeruginosa	1	1	3	2	2
K. pneumoniae	32	4	4	1	4

Figure S1: Distribution of subtype of carbapenem-resistance genes in their respective groups of GNB isolates

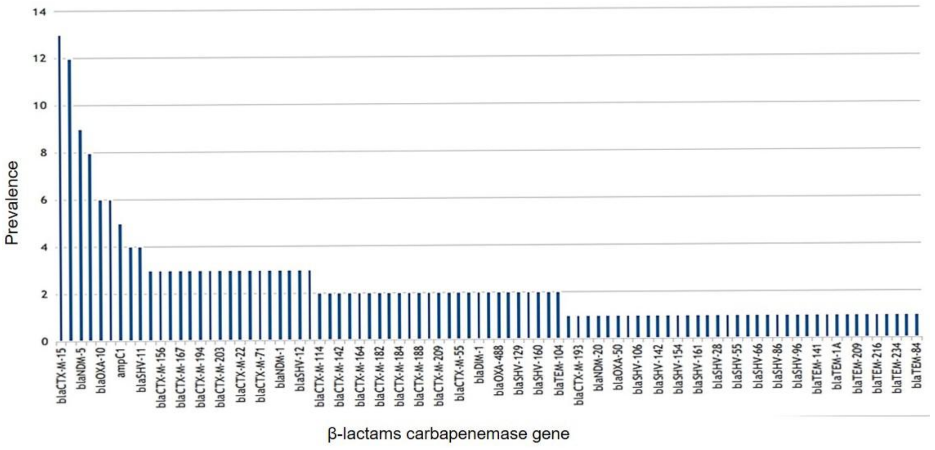

Supplement: Supplementary file 1 — Supplementary Information. [file 41598_2022_12471_MOESM1_ESM.pdf]
